# Risk Factors for Internet Gaming Disorder: Psychological Factors and Internet Gaming Characteristics

**DOI:** 10.3390/ijerph15010040

**Published:** 2017-12-27

**Authors:** Mi Jung Rho, Hyeseon Lee, Taek-Ho Lee, Hyun Cho, DongJin Jung, Dai-Jin Kim, In Young Choi

**Affiliations:** 1 Department of Medical Informatics, College of Medicine, The Catholic University of Korea, Seoul 06591, Korea; rhomijung@gmail.com; 2 Catholic Institute for Healthcare Management and Graduate School of Healthcare Management and Policy, The Catholic University of Korea, Seoul 06591, Korea; 3 Department of Industrial & Management Engineering, Pohang University of Science and Technology, Pohang 37673, Korea; hyelee@postech.ac.kr (H.L.); dlxorgh2@postech.ac.kr (T.-H.L.); 4 Department of Psychology, Korea University, Seoul 02841, Korea; sonap1@hanmail.net; 5 Addiction Research Institute, Department of Psychiatry, Seoul St. Mary’s Hospital, College of Medicine, The Catholic University of Korea, Seoul 06591, Korea; forever0851@naver.com; 6 Department of Psychiatry, Seoul St. Mary’s Hospital, College of Medicine, The Catholic University of Korea, Seoul 06591, Korea

**Keywords:** internet gaming disorder, Dickman Impulsivity Inventory-Short Version (DII), Brief Self-Control Scale (BSCS), Symptom Checklist-90-Revised (SCL-90-R), Behavioral Inhibition System/Behavioral Activation System (BIS/BAS), Diagnostic and Statistical Manual for Mental Disorders (DSM-5)

## Abstract

*Background*: Understanding the risk factors associated with Internet gaming disorder (IGD) is important to predict and diagnose the condition. The purpose of this study is to identify risk factors that predict IGD based on psychological factors and Internet gaming characteristics; *Methods*: Online surveys were conducted between 26 November and 26 December 2014. There were 3568 Korean Internet game users among a total of 5003 respondents. We identified 481 IGD gamers and 3087 normal Internet gamers, based on Diagnostic and Statistical Manual for Mental Disorders (DSM-5) criteria. Logistic regression analysis was applied to identify significant risk factors for IGD; *Results*: The following eight risk factors were found to be significantly associated with IGD: functional and dysfunctional impulsivity (odds ratio: 1.138), belief self-control (1.034), anxiety (1.086), pursuit of desired appetitive goals (1.105), money spent on gaming (1.005), weekday game time (1.081), offline community meeting attendance (2.060), and game community membership (1.393; *p* < 0.05 for all eight risk factors); *Conclusions*: These risk factors allow for the prediction and diagnosis of IGD. In the future, these risk factors could also be used to inform clinical services for IGD diagnosis and treatment.

## 1. Introduction

Since Internet games became widespread in the 2000s [1], Internet game usage has experienced rapid growth among both youth and adults. According to a report by the Entertainment Software Association (ESA) [2], 155 million Americans play video games, of which 42% play video games regularly. In 2015 alone, American game consumers spent more than US$22.41 billion on game content, hardware, and accessories [2]. Worldwide Internet game usage and gaming money has been rapidly increasing. As a result, Internet Gaming Disorder (IGD) has become a major social problem and important research topic. The World Health Organization (WHO) has proposed a new category named “Gaming Disorder” for the 11th Revision of the International Classification of Diseases (ICD-11) [3]. The ability to predict, diagnose, and manage IGD in advance is critical to the prevention of IGD. To do that, the risk factors associated with IGD need to be better understood. 

Firstly, the psychological factors associated with IGD need to be understood. IGD can be considered a behavioral addiction [4,5,6,7,8] and has been found to be related to a number of psychological and health problems, including depression, social anxiety, fatigue, loneliness, negative self-esteem, and impulsivity [9,10,11,12]. IGD co-occurs with various psychiatric conditions and can lead to a range of negative outcomes. For example, IGD can cause social problems such as lower academic achievement [10,11,13,14,15,16,17]. In addition, IGD shares many similarities with other addictions, such as substance use disorder [18]. 

Secondly, the Internet gaming characteristics associated with IGD need to be better understood. Research in this area has increased in both quantity and quality. In order to predict, diagnose, and manage IGD, researchers have attempted to identify the causes and negative consequences of excessive gaming as well as risk factors of IGD. Some research, however, has only focused on psychological factors [16,19] or Internet gaming characteristics, such as the level of Internet usage, money spent on gaming, and type of game device [20]. A comprehensive approach based on both psychological factors and Internet gaming characteristics is needed to better understand IGD. Accordingly, the purpose of the present study was to identify risk factors that predict IGD, based on psychological factors and Internet gaming characteristics.

## 2. Materials and Methods

### 2.1. Participants

Online surveys were conducted using an existing survey company online panel (Hankook Research, Inc., Seoul, South Korea between 26 November and 26 December 2014. Online informed consent was obtained from all participants, prior to their participation. The online panel consisted of native Koreans aged 20–49 years, from metropolitan areas in South Korea. Among a total of 5003 respondents, 3881 Internet game users were identified. The final sample size comprised 3568 Internet game users, which did not include missing values.

Using the DSM-5 criteria to diagnose IGD is controversial [3,21]. Some researchers have attempted to overcome this confusion [21,22]. Because there are very few criteria for IGD in the DSM-5, it was used to evaluate IGD in the present study. In addition, DSM-5 criteria were validated from discussions among an expert group. Based on DSM-5 criteria, Internet game users with scores above 5 were evaluated as the IGD group [20,23]. Thus, in the final sample, there were 481 IGD gamers (13.48% of the sample) and 3087 normal Internet gamers (86.52%).

### 2.2. Measures and Procedure

Twenty independent variables were measured as potential risk factors for IGD. Independent variables consisted of participants’ demographic characteristics, Internet gaming characteristics, and psychological variables.

In the case of Internet gaming characteristics, there were very few related studies, so related variables could not be chosen from the existing literature. Internet gaming characteristics were therefore derived from the Internet Addiction Survey 2013 conducted by the Korea National Information Society Agency [24]. The specific items were identified from discussions among an expert group. The expert group consisted of psychiatrists, psychologists, and data scientists of medical informatics who had more than 3 years’ experience in addiction. Psychological variables were derived from previous research and were again collected from discussions among an expert group. The reliability of all variables was determined by the expert group.

Participants’ demographic characteristics consisted of five factors: gender, age, job, score on the Alcohol Use Disorder Identification Test (AUDIT-K) [25], and score on the Fagerström Test for Nicotine Dependence (FTND) [26]. Participant data were divided into three groups based on AUDIT-K and FTND scores, as summarized in Appendix A (Table A1). The AUDIT-K is a ten-item questionnaire developed for male and female drinkers at a high risk of alcohol abuse. It is composed of three scores that are dependent on gender: male (0–9: normal drinker, 10–19: mild-to-moderate drinker, and ≥20: heavy drinker) and female (0–5: normal drinker, 6–9: mild-to-moderate drinker, and ≥10: heavy drinker). The FTND test is a six-item questionnaire designed to measure nicotine dependence. It is composed of three scores (0–3: low, 4–6: intermediate, and ≥7: high).

Seven Internet gaming characteristics were also measured: money spent on gaming, weekday game time, weekend game time, game device, game venue, offline game club attendance, and game club membership status.

Finally, eight psychological variables were measured, including the Dickman Impulsivity Inventory-Short Version (DII), Brief Self-Control Scale (BSCS) [27], Symptom Checklist-90-Revised (SCL-90-R) [28] and Behavioral Inhibition System/Behavioral Activation System (BIS/BAS) [29,30], as summarized in Table 1. The DII measures the personality trait of impulsivity [31]. The response options for each item are true (1) or false (0). The BSCS assesses dispositional self-control [27]. Each BSCS item is rated on a five-point scale, from 1 (strongly disagree) to 5 (strongly agree). The SCL-90-R consists of 90 items and assesses psychological distress [32,33]. Each of the items is rated on a five-point scale of distress, from 0 (no distress) to 4 (extreme distress). In the present study, 23 items from the SCL-90-R were adapted to evaluate depression (13 items) and anxiety (10 items). 

The behavioral inhibition system (BIS) and a behavioral activation system (BAS) underlie behavior and affect [30]. The BIS scale estimates reactions to anticipated punishment and the BAS scale assesses positive responses to rewards. The BAS Drive scale estimates the pursuit of desired goals. The BAS Fun Seeking scale examines the tendency to seek and impulsively engage in potentially rewarding activities [30,34]. The BIS/BAS consists of a four-point scale, from 1 (not at all) to 4 (strongly agree). The total scores of the BIS/BAS scales range from zero to 80.

Questions related to cost, gaming time, and age were self-reported questions and free text which yielded a continuous value. The rest were multiple choice questions based on predefined categories.

### 2.3. Statistical Analysis

Out of 3881 respondents who identified as Internet game users, cases with missing responses were excluded, and all analyses were performed for 3568 respondents. We conducted *t*-tests and Chi-square tests to compare the IGD group to the control group in terms of demographic and Internet gaming characteristics. Multiple regression analysis was used to identify risk factors for the IGD group. The data were analyzed using SAS 9.4 (SAS Institute, Inc., Cary, NC, USA).

### 2.4. Ethics

The study procedures were carried out in accordance with the Declaration of Helsinki and were approved by the Institutional Review Board of Catholic University (IRB number: KC15EISI0103). Participants’ data were de-identified.

## 3. Results

Out of 3568 participants, 481 (13.5%) were included in the IGD group and 3087 (86.5%) were included in the control group. The respondents’ age ranged from 20 to 49, and 1559 (43.7%) were between the ages of 30 and 39. There were 2036 (57.1%) males and 1532 (42.9%) females (Table 2). Office workers and professional technicians comprised 67.8% of the sample, and college students comprised 15%. There were similar proportions of individuals in each group with a marital status of either single or married. There were no significant differences in demographic characteristics between the two groups; however, males were more likely to be in the IGD group than females. For income level, there were more people from the control group in the middle class, while low and high income classes showed slightly higher dependence.

Differences in Internet gaming characteristics for all variables except game playing were significant between the IGD group and the control group (Table 3). Among all participants, 57.8% of the IGD group had a game club membership, while 35.4% of the control group had a game club membership. The respondents having a game club membership showed higher IGD than the control group (57.8% vs. 35.4%). Most of the Internet game users played at home, and there was no difference between the IGD group and control group (76.1% vs. 77.2%). In the case of playing in a gaming Internet cafe, the IGD group was much higher than the control group (17.5% vs. 10.2%). For game devices, the IGD group used a personal computer (PC) more than the control group (53.0% vs. 37.9%). For game partners, those playing with friends or online partners showed higher dependence than the control group (29.1% vs. 21.5%). Both the IGD and the control group had perceptions of addictiveness. For offline club game attendance, the IGD group’s attendance was much higher than that of the control group (57.3% vs. 26.6%). For the onset of Internet games, 48.3% of respondents began in middle or high school. The IGD group spent more time gaming than the control group (2.85 vs. 1.97 h on weekdays and 4.12 vs. 2.92 h on weekends, respectively) and spent more money on gaming than the control group ($31.4 vs. $11.0, respectively).

### Risk Factors Predicting IGD

The results of the multivariate logistic regression analysis are shown in Table 4. Firstly, demographic characteristics were shown not to be risk factors. All variables included in the logistic regression model do not show muliticollinearity. Secondly, with regard to Internet gaming characteristics, money spent on gaming (OR = 1.005), weekday game time (OR = 1.081), offline game club attendance (OR = 2.060), and game club membership status (OR = 1.393) were significant behavioral factors predicting IGD. Thirdly, DII (OR = 1.138), BSCS (OR = 1.034), anxiety (OR = 1.086), and BAS-Drive (OR = 1.105) were significant psychological predictors of IGD. Those who had one unit score higher for DII were 1.138 times more likely to be dependent. Additionally, with one unit score higher for the BSCS, Anxiety, and BAS-Drive factors, the probability of dependence increased by 1.034, 1.086, and 1.105 times, respectively. One of measures for model performance in a general linear model, Nagelkerke’s R^2^ is 0.3012 which showed it was a better model than others [35].

## 4. Discussion

We identified risk factors predicting IGD, specifically examining psychological and Internet gaming characteristics as potential risk factors. Based on the results of the present study, we draw the following conclusions.

Firstly, examination of psychological factors yielded meaningful results. Users with IGD perceived themselves as being obsessed with Internet gaming (Table 3) and that they had difficulty quitting the game. Thus, social support may be needed to prevent IGD and support treatment efforts. Psychological risk factors related to IGD included impulsivity, low self-control, anxiety, and pursuit of desired appetitive goals. Past research has shown that IGD has similarities to other addictions, such as gambling and substance use disorder [18,36,37]. In particular, impulsivity and self-control are important psychological factors affecting addiction [38,39]. Impulsivity has been reported as a risk factor in addiction to social networking sites or smartphones [29,40] and lack of self-control is related to addictions such as substance use disorder [27] and Internet use [41,42,43]. Anxiety may be relevant psychopathological symptom to detect Internet, smartphone, and video game addiction [44,45,46]. Lastly, BAS Drive was a risk factor associated with IGD. The level of BAS Drive represents the tendency to pursue desired goals actively [34] and has been shown to be one of the personality factors associated with smartphone addiction [29]. This shows that to predict and diagnose IGD, research on the associated psychological risk factors is needed.

Secondly, a number of Internet gaming characteristics were significant in predicting IGD. Users with IGD mainly played games at home. In the case of playing games in a gaming Internet cafe, the proportion of individuals with dependence was higher than normal (17.5% vs. 10.2%). Game users mainly played using a PC compared to a mobile device (53.0% vs. 37.9%) since high specification desktops were needed. However, the control group played games more frequently using mobile devices compared to PCs. With regard to the onset of Internet gaming, 48.3% of respondents began in middle or high school. Users with IGD tended to start playing Internet games at a relatively early age. This finding suggests that early initiation of game playing may be a risk factor for IGD. Accordingly, diverse approaches are needed early on to prevent adolescent and adult IGD. Offline game club attendance and game club membership status were also risk factors for IGD. Users with IGD were more likely to be game club members than those in the control group (57.3% vs. 26.6%) and were more likely to attend offline clubs, with 73.3% of the control group having never attended offline game meetings. On average, users with IGD were thought to have no social relationships and to be more isolated. However, they did attend offline game clubs and have game club memberships. There were some social users with IGD.

Additional risk factors of IGD were money spent on gaming and weekday game time. In the case of game time, Internet game users spent an average of 2.09 h on weekends playing games. Users with IGD spent more time than normal gamers playing Internet games (2.85 vs. 1.97 h on weekdays and 4.12 vs. 2.92 h on weekends). According to the Ministry of Science ICT and Future Planning (MSIP) report, Korean gamers spent an average of 1.1 h on weekends playing games. Users with Internet over-dependence spent 0.3 more hours playing on weekends than normal users (1.4 vs. 1.1 h) [47]. The results from our study show that game time was higher in our sample. The MSIP report focused on individuals ranging in age from early childhood (3 years) to 59 years whereas our results came from a sample of adults between the ages of 20 and 49. This higher game time suggests that IGD is more serious in adults. Users with IGD spent more money on gaming than the control group ($31.4 vs. $11.0). Previous research has reported that spending extreme amounts of time and money is a predictor of IGD [20,48,49,50,51,52]. Lo et al., (2005) found that the amount of time spent playing online games is directly correlated with levels of social anxiety [50]. Rau et al., (2006) proposed that many game players have difficulty in controlling game time [49]. Accordingly, approaches are needed for IGD among adults and controlling time and money is important to preventing and managing IGD.

## 5. Conclusions

This study had several limitations. Data on Internet gaming characteristics were self-reported, including money spent on gaming, weekday game time, and weekend game time. If technology could be developed, such as the Smartphone Overdependence Management System (SOMS), to collect time or money data automatically [53], future research may provide more accurate and realistic results. We collected data using an online survey. This was based on an existing online panel from a survey company. Online panel respondents were native Koreans aged 20–49 years, from metropolitan areas in South Korea. Using an online survey based on an existing panel was a useful way to collect a large amount of data; however, this may have resulted in some recruitment bias. Future research should involve data collected from the entire Korea area. The present study was designed to be cross-sectional because it is difficult to collect time-series data from Internet gamers. As a result, our findings are limited in their ability to reflect fast-changing Internet gaming trends. Future research could incorporate time-series data from longitudinal studies. Future research could also involve a more accurate diagnosis of IGD based on a clinical interview. The results showed that depression has no significant relationship with IGD. This is contrary to other published studies that have found video or internet game addiction to be related to depression [10,45,54]. We used the SCL-90-L to evaluate depression; however, there are many other scales to measure depression, such as the 21-item Depression Anxiety Stress Scale (DASS-21) [55] and the Hopkins Symptom Checklist (HSCL) [56]. Future studies should evaluate depression using other measures. This study targeted respondents aged between 20 and 49, of which 1559 (43.7%) were between the ages of 30 and 39. Therefore, the reported results could be influenced by demographic characteristics.

Despite these limitations, the present study yielded a valuable contribution to our understanding of risk factors for IGD by using a comprehensive approach based on psychological factors and Internet gaming characteristics. These findings can be used to develop clinical services for the diagnosis and treatment of IGD.

## Figures and Tables

**Table 1:** Description of Internet gaming characteristics and psychological factors.

Variables		# of Items
Demographic characteristics	Gender, age, job	3
	AUDIT-K	10
	FTND	6
Internet gaming characteristics	Money spent on gaming (/month), Weekday game time (/day), Weekend game time (/day), Game device, Game venue, Offline game club attendance, Game club membership status	7
Psychological factors	DII	12
	BSCS	13
	SCL depression	13
	SCL anxiety	10
	BIS	7
	BAS reward responsiveness	5
	BAS drive	4
	BAS fun seeking	4

AUDIT-K: Alcohol Use Disorder Identification Test; FTND: Fagerström Test for Nicotine Dependence; DII: Dickman Impulsivity Inventory-Short Version; BSCS: Brief Self-Control Scale; SCL: Symptom Checklist; BIS: behavioral inhibition system; BAS: behavioral activation system.

**Table 2:** Participants’ characteristics.

Variables		Total	IGD Group	Control Group	Chi-Square (*p*-Value)
		*n* (%)	*n* (%)	*n* (%)	
Gender	Mal	2036 (57.1)	290 (60.3)	1746 (56.6)	2.36 (0.124)
	Female	1532 (42.9)	191 (39.7)	1341 (43.4)	
Age	20–29 years	1259 (35.3)	170 (35.3)	1089 (35.3)	0.43 (0.808)
	30–39 years	1559 (43.7)	215 (44.7)	1344 (43.5)	
	40–49 years	750 (21.0)	96 (20.0)	654 (21.2)	
Education	High school graduate or less	1053 (29.5)	134 (27.9)	919 (29.8)	0.76 (0.683)
	College graduate	2130 (59.7)	295 (61.3)	1835 (59.4)	
	Graduate school	385 (10.8)	52 (10.8)	333 (10.8)	
Job	Office worker, et al. ^1^	2418 (67.8)	334 (69.4)	2084 (67.5)	0.86 (0.835)
	Student	535 (15.0)	67 (13.9)	468 (15.2)	
	etc.	217 (6.1)	27 (5.6)	190 (6.2)	
	Unemployed/housewife	398 (11.2)	53 (11.0)	345 (11.2)	
Marital status	Couple ^2^	1867 (52.3)	241 (50.1)	1626 (52.7)	1.10 (0.294)
	Single ^2^	1701 (47.7)	240 (49.9)	1461 (47.3)	
Income level	Low	1567 (43.9)	219 (45.5)	1348 (43.7)	3.52 (0.172)
	Middle	1557 (43.6)	193 (40.1)	1364 (44.2)	
	High	444 (12.4)	69 (14.3)	375 (12.2)	
Total		3568 (100)	481 (13.5)	3087 (86.5)	

^1^ Office worker et al.: office worker, administrative position, service industry, professional technician and production employee; ^2^ Single: never married, divorced, separated or widowed, Couple: married or living with a partner; IGD: Internet gaming disorder.

**Table 3:** Internet gaming characteristics.

Variables		Total	IGD Group	Normal Group	Test Statistics (*p*-Value)
		*n* (%)	*n* (%)	*n* (%)	
Game club membership	No	2198 (61.6)	203 (42.2)	1995 (64.6)	88.45 (<0.001)
	Yes	1370 (38.4)	278 (57.8)	1092 (35.4)	
Game playing	Playing one game intensively	2098 (58.8)	302 (62.8)	1796 (58.2)	3.65 (0.056)
	Playing various games	1470 (41.2)	179 (37.2)	1291 (41.8)	
Game venue	Home	2748 (77.0)	366 (76.1)	2382 (77.2)	32.85 (<0.001)
	Gaming Internet cafe	400 (11.2)	84 (17.5)	316 (10.2)	
	Others ^1^	420 (11.8)	31 (6.4)	389 (12.6)	
Game device	PC	1424 (39.9)	255 (53.0)	1169 (37.9)	42.39 (<0.001)
	Console	63 (1.8)	11 (2.3)	52 (1.7)	
	Mobile device ^2^	2080 (58.3)	215 (44.7)	1865 (60.4)	
Game partner	Alone	2593 (72.7)	321 (66.7)	2272 (73.6)	14.07 (0.003)
	Family	169 (4.7)	20 (4.2)	149 (4.8)	
	Friends	280 (7.9)	52 (10.8)	228 (7.4)	
	Online partner	526 (14.7)	88 (18.3)	438 (14.2)	
Self-perceptions of addictiveness	Not at all	203 (5.7)	19 (4.0)	184 (6.0)	85.69 (<0.001)
	A little	1077 (30.2)	90 (18.7)	987 (32.0)	
	Much	1979 (55.5)	285 (59.3)	1694 (54.9)	
	Very much	309 (8.7)	87 (18.1)	222 (7.2)	
Offline game club attendance	Not attend	2469 (69.2)	205 (42.6)	2264 (73.3)	185.63 (<0.001)
	Sometimes	1032 (28.9)	256 (53.2)	776 (25.1)	
	Very often	67 (1.9)	20 (4.2)	47 (1.5)	
Onset of Internet game	Under middle school	842 (23.6)	122 (25.4)	720 (23.3)	11.42 (0.009)
	Middle or high school	882 (24.72)	142 (29.5)	740 (24.0)	
	After graduating high school	1056 (29.6)	131 (27.2)	925 (30.0)	
	30s or 40s	788 (22.09)	86 (17.9)	702 (22.7)	
Gaming time/day	Weekdays	2.09	2.85	1.97	7.21 (<0.001)
	Weekends and holidays	3.08	4.12	2.92	7.19 (<0.001)
	Maximum	4.07	5.93	3.78	6.30 (<0.001)
Money spent on gaming/month		$13.76	$31.36	$11.02	8.23 (<0.001)

Time unit: hours, the exchange rate for Korean won to the U.S. dollar is 1100.00 won (September 2016), t-statistics for continuous variable, and chi-square value for categorical variables. ^1^ Others: School, play station room, the outside including bus, substation; ^2^ Mobile device: Smartphone and Tablet.

**Table 4:** Risk factors predicting IGD.

Variables		Estimate (SE)		*p*-Value	OR 95% CI
Intercept		−5.452 (0.602)			**-**
Gender		0.023 (0.139)		0.869	1.023 (0.779–1.344)
Age		0.138	0.090	0.125	1.148 (0.962–1.37)
Job	Office worker, et al. ^1^	−0.167	0.193	0.387	0.846 (0.579–1.236)
	Student	−0.017	0.248	0.944	0.983 (0.604–1.599)
	etc.	−0.260	0.291	0.373	0.771 (0.436–1.365)
AUDIT	Normal drinker	−0.136	0.163	0.404	0.873 (0.634–1.201)
	Mild-to-moderate drinker	−0.313	0.166	0.059	0.731 (0.528–1.012)
	Heavy drinker	0.171	0.161	0.289	1.186 (0.865–1.626)
FTND	Low	−0.201	0.171	0.240	0.818 (0.585–1.144)
	Intermediate	0.177	0.195	0.362	1.194 (0.815–1.748)
	High	0.358	0.307	0.243	1.431 (0.784–2.611)
Money spent on gaming ***		0.005	0.002	<0.001 ***	1.005 (1.002–1.008)
Weekday game time ***		0.078	0.027	0.003 ***	1.081 (1.026–1.139)
Weekend game time		0.004	0.019	0.843	1.004 (0.968–1.041)
Game device	PC	0.160	0.132	0.224	1.174 (0.907–1.519)
	Console	0.239	0.413	0.563	1.270 (0.565–2.853)
Game venue	Home	0.324	0.217	0.135	1.383 (0.905–2.114)
	Gaming Internet cafe	0.282	0.270	0.296	1.326 (0.781–2.25)
Offline game club attendance ***		0.723	0.130	<0.001 ***	2.060 (1.597–2.658)
Game club membership status **		0.332	0.125	0.008 **	1.393 (1.09–1.78)
DII ***		0.129	0.022	<0.001 ***	1.138 (1.09–1.188)
BSCS **		0.034	0.012	0.006 **	1.034 (1.01–1.059)
SCL Depression		−0.008	0.012	0.496	0.992 (0.968–1.016)
SCL Anxiety ***		0.082	0.015	<0.001 ***	1.086 (1.054–1.118)
BIS		−0.031	0.025	0.215	0.969 (0.923–1.018)
BAS reward responsiveness		0.005	0.039	0.908	1.005 (0.93–1.085)
BAS drive *		0.100	0.041	0.015 *	1.105 (1.02–1.198)
BAS fun seeking		−0.063	0.042	0.133	0.939 (0.865–1.019)

SE: standard error; * *p* < 0.1, ** *p* < 0.05, *** *p* < 0.01; ^1^ Office worker, et al.: office worker, administrative position, service industry, professional technician and Production employee.

## Appendix A

**Table A1:** Criteria and score in the AUDIT and the FTND tests.

AUDIT Test			FTND Test	
Category	Male	Female		
	Score		Category	Score
Normal drinker	≤9	≤5	Low risk	≤3
Mild-to-moderate drinker	10~19	6~9	Intermediate risk	4~6
Heavy drinker	≥20	≥10	High risk	≥7
